# Prognostic factors and predictive model construction in patients with non-small cell lung cancer: a retrospective study

**DOI:** 10.3389/fonc.2024.1378135

**Published:** 2024-05-24

**Authors:** Shixin Ma, Lunqing Wang

**Affiliations:** ^1^ Dalian Medical University, Dalian, Liaoning, China; ^2^ Department of Thoracic Surgery, Qingdao Municipal Hospital, Qingdao, Shandong, China

**Keywords:** non-small cell lung cancer, overall survival, progression free survival, prediction model, prognosis

## Abstract

**Objective:**

The purpose of this study was to construct a nomogram model based on the general characteristics, histological features, pathological and immunohistochemical results, and inflammatory and nutritional indicators of patients so as to effectively predict the overall survival (OS) and progression-free survival (PFS) of patients with non-small cell lung cancer (NSCLC) after surgery.

**Methods:**

Patients with NSCLC who received surgical treatment in our hospital from January 2017 to June 2021 were selected as the study subjects. The predictors of OS and PFS were evaluated by univariate and multivariable Cox regression analysis using the Cox proportional risk model. Based on the results of multi-factor Cox proportional risk regression analysis, a nomogram model was established using the R survival package. The bootstrap method (repeated sampling for 1 000 times) was used to internally verify the nomogram model, and C-index was used to represent the prediction performance of the nomogram model. The calibration graph method was used to visually represent its prediction compliance, and decision curve analysis (DCA) was used to evaluate the application value of the model.

**Results:**

Univariate and multivariate analyses were used to identify independent prognostic factors and to construct a nomogram of postoperative survival and disease progression in operable NSCLC patients, with C-index values of 0.927 (907–0.947) and 0.944 (0.922–0.966), respectively. The results showed that the model had high predictive performance. Calibration curves for 1-year, 2-year, and 3-year OS and PFS show a high degree of agreement between the predicted probability and the actual observed probability. In addition, the results of the DCA curve show that the model has good clinical application value.

**Conclusion:**

We established a predictive model of survival prognosis and disease progression in patients with non-small cell lung cancer after surgery, which has good predictive performance and can guide clinicians to make the best clinical decision.

## Introduction

According to the findings of the Global Cancer Epidemiological Survey, it is projected that there will be a total of 19.3 million newly diagnosed cases of cancer and 9.9 million deaths attributed to cancer globally in the year 2020. On a global scale, it is observed that China contributes to approximately 24% of newly diagnosed cancer cases and approximately 30% of cancer-related mortalities. Lung cancer continues to be the prevailing form of cancer in China and the primary contributor to mortality associated with cancer (1). About 85% of people who are diagnosed with lung cancer are told they have non-small cell lung cancer (NSCLC). Lung adenocarcinoma (LUAD) and lung squamous cell carcinoma (LUSC) are the most common subtypes of NSCLC (2). Regrettably, a significant majority of individuals who receive a diagnosis of lung cancer exhibit a dearth of distinct symptoms during the initial phases of the illness. Patients who present with symptoms such as hemoptysis, chest discomfort, and chest tightness typically indicate an advanced stage of the disease, rendering them ineligible for surgical intervention. Consequently, the prognosis for these patients is characterized by a notably poor 5-year survival rate (3). During the initial phases of non-small cell lung cancer, surgical intervention remains the predominant therapeutic approach, with a potential 5-year survival rate above 80% following the surgical procedure (4, 5). Nevertheless, it is important to acknowledge that surgical intervention continues to pose potential hazards such as recurrence, metastasis, and mortality. It is noteworthy that a significant proportion, exceeding 50%, of recurrences and metastases manifest in patients within a two-year timeframe subsequent to the surgical procedure (6). So, using sensitive predictive markers before treatment lets doctors figure out how the therapy will work and what the patient’s outlook is. When figuring out how to treat NSCLC, a number of prognostic factors are used. The American Joint Committee on Cancer (AJCC) tumor nodule metastasis (TNM) stage is one of the most important (7). Still, the chance of survival for people with a certain stage of NSCLC varies a lot depending on things like age, gender, smoking habits, how many lymph nodes are removed during surgery, the histological features seen after surgery, treatment plan, and other similar factors (8). These factors collectively influence the individual survival outcomes of NSCLC patients (9, 10). The nomogram model serves as a graphical computing tool that combines the significant pathological attributes of the tumor with the clinical parameters of the patient in order to forecast the prognosis. The assessment of cancer risk by this method is universally regarded as reliable and is extensively employed in clinical research (11, 12). The objective of this study was to conduct a comprehensive analysis of the various factors that influence the survival prognosis and disease progression in patients with operable NSCLC. These factors include baseline characteristics, histological features, pathological and immunohistochemical results, inflammation, and nutritional indexes. The aim was to provide valuable insights and references for clinical practice.

## Material and method

### object of study

Patients with NSCLC who received surgical treatment in our hospital from January 2017 to June 2021 were selected as the study subjects. All patients included in this study met the following criteria: (1) patients had to be at least 18 years old; (2) they had to have thoracoscopic surgery and were pathologically confirmed to have non-small cell lung cancer (stages I–III according to the 8th TNM edition) (13); (3) they had no history of malignant tumors or a second primary cancer; and (4) full preoperative clinical data were available. Exclusion criteria: (1) NSCLC patients who are unresectable or cannot tolerate surgical treatment; (2) patients with blood system diseases, immune system diseases, or blood abnormalities of unknown cause. (3) There are serious underlying diseases in the past (such as grade IV heart function, liver and kidney failure, stroke with serious sequelae, etc.), resulting in unclear outcome indicators; (4) there is incomplete clinical data or incomplete follow-up records.

### Study design

The present investigation was conducted as a retrospective case-control study. The study protocol received approval from the Institutional Review Committee of Qingdao Municipal Hospital, and the study method was conducted in strict adherence to the principles outlined in the Declaration of Helsinki.

### Data collection

The hospital’s electronic medical record filing system, quality control registration management system, and laboratory examination reporting system were used to collect information on patients in four areas: baseline characteristics, histological characteristics, pathological characteristics, and nutrition and inflammation indicators. The collected data encompassed variables such as age, gender, smoking status, and body mass index (BMI). The variables considered in this study include the Eastern Cooperative Oncology Group Performance Status (ECOG PS) score, past medical history related to the respiratory system, adjuvant therapy such as chemotherapy and radiotherapy, preoperative pulmonary function indicators, tumor location, surgical plan, TNM stage, immunohistochemical and inflammatory indicators such as neutrophils, lymphocytes, monocytes, C-reactive protein, platelets, serum free fatty acids, globulins, alkaline phosphatase, and fibrinogen. Additionally, nutritional status indicators, including albumin, prealbumin, and erythrocyte distribution width, were taken into account. Patient survival status was also considered an outcome measure. The BMI is categorized into three groups: underweight (< 18.5 kg/m^2^), normal weight (18.5-23.9 kg/m^2^), and obese (≥ 24 kg/m^2^). The primary parameters used to assess lung function are forced vital capacity (FVC) expressed as a percentage, forced expiratory volume in the first second (FEV_1_) expressed as a percentage, and the ratio of FEV_1_ to FVC expressed as a percentage. In light of the absence of a universally accepted standard, this research employed a categorization approach that relied on the crucial values of FEV_1_ (%) and FEV_1_/FVC (%) as well as the findings of prior investigations (14). To adhere to the stipulation that the sample size for the multi-factor analysis should be at least 10–20 times greater than the number of independent variables, it is recommended to incorporate a sample of 400–800 participants into the study. In light of potential limitations such as loss of follow-up and other cases, it is recommended to augment the initial sample size by 10%, resulting in a final range of 440–880 (15). According to the inclusion and exclusion criteria, the final number of patients included in this study was 899.

### Follow up

The patients were followed up according to the hospital’s outpatient and inpatient medical records system and telephone form, and the deadline for follow-up was December 2022. The date of pathological diagnosis of NSCLC was first defined as time zero; the death and disease progression of lung cancer were the outcome events; and the survival at the end of follow-up was the truncated event. Overall survival (OS) is a final or truncated event from zero in time until death occurs; progression-free survival (PFS) is an outcome event that begins at zero point in time until disease progression occurs. The median follow-up was 34.2 months.

### Statistical method

SPSS 27.0 software and R 4.2.1 software were used for data processing and analysis. The Kolmogorov-Smirnov method was used to test the normality of the measurement data. Those who met the normal distribution were represented by the positive and negative standard deviation of the mean, and a *T*-test was used for comparison between groups. Those who do not conform to the normal distribution are represented by Median (1st quartile, 3rd quartile), and the Mann-Whitney U test is applied for inter-group comparison. The count data were represented by the number of cases (%), and the comparison between groups was performed by a chi-square test or Fisher exact test. Survival analysis was presented by the Kaplan-Meier curve, and differences between groups were compared by the log-rank test.

The best cutoff values of tumor diameter, inflammation, and nutrition complex indexes were obtained by the receiver operator characteristic (ROC) curve and were divided into two categorical variables. The predictors of OS and PFS were evaluated by univariate and multivariable Cox regression analysis using the Cox proportional risk model. Based on the results of multi-factor Cox proportional risk regression analysis, a nomogram model was established using the R survival package. The prediction performance of the nomogram model was verified by the Bootstrap method (repeated sampling 1 000 times), and the concordance index (C-index) was used to represent the prediction performance of the nomogram model, and the calibration graph method was used to directly represent its prediction conformity. *P*<0.05 was considered statistically significant.

## Results

### Univariate Cox regression analysis

#### Correlation analysis based on baseline features

This study conducted a comprehensive review of patients with NSCLC who underwent surgery in our hospital between January 2017 and June 2021, and a total of 899 patients were included in the analysis based on predetermined inclusion and exclusion criteria. The median age of the patients was 62 ± 9.64 years, and the age range was 25–89 years. 54.1% of the patients were female. The mean BMI of the patients was 24.67 ± 3.38. 283 patients (about 31.5% of the sample) had a history of smoking. 220 patients had a history of respiratory system disease, accounting for 24.5% of the samples. In addition, 170 patients (18.9% of the sample) presented with an ECOG PS score of 2 or above. In the follow-up survival assessment of patients, 71 (7.9%) died of lung cancer and 133 (14.8%) developed disease progression within 3 years after surgery. The rates of OS and PFS at 1, 2, and 3 years were 98.2%, 95.3%, and 92.10%, and 95.2%, 89.1%, and 85.2%, respectively.

Univariate analysis of patients’ baseline characteristics showed that female patients and patients with FVC≥80% had a better prognosis; smoking history and adjuvant therapy were significant factors for poor survival after surgery. See **Table 1**. In the PFS association analysis, patients ≥65 years of age, a history of smoking, and adjuvant therapy were more likely to have disease progression, while women, FEV_1_, and FVC≥80% were associated with improvements in PFS (**Table 1**).

**Table 1:** Univariate Cox regression analysis based on baseline characteristics.

Category	n (%)	Overall Survival			Progression Free Survival		
		*HR*	*95% CI*	*P - value*	*HR*	*95% CI*	*P - value*
Age (years)							
<65	561 (62.4)		1.000			1.000	
≥65	338 (37.6)	1.358	0.908∼2.031	0.136	1.426	1.032∼1.969	0.032
Gender							
Male	413 (45.9)		1.000			1.000	
Female	486 (54.1)	0.593	0.395∼0.891	0.012	0.536	0.385∼0.745	<0.001
Smoking status							
Never smoked	616 (68.5)		1.000			1.000	
Current/past smoking	283 (31.5)	1.885	1.264∼2.812	0.002	2.287	1.658∼3.156	<0.001
BMI (kg/m²)							
<18.5	22 (2.4)		1.000			1.000	
18.5∼23.9	366 (40.7)	2.324	0.321∼16.826	0.404	0.916	0.336∼2.498	0.864
>24	511 (56.8)	3.135	0.432∼22.765	0.259	1.017	0.370∼2.798	0.974
ECOG PS score							
0 - 1	729 (81.1)		1.000			1.000	
≥2	170 (18.9)	1.195	0.730∼1.955	0.479	1.331	0.908∼1.952	0.143
disease of respiratory system							
No	679 (75.5)		1.000			1.000	
Yes	220 (24.5)	1.401	0.910∼2.157	0.126	1.256	0.878∼1.795	0.212
Adjuvant chemotherapy							
No	555 (61.7)		1.000			1.000	
Yes	344 (38.3)	38.595	12.167∼122.426	<0.001	39.898	17.600∼90.444	<0.001
Adjuvant radiation therapy							
No	854 (95.0)		1.000			1.000	
Yes	45 (5.0)	6.865	4.364∼10.801	<0.001	11.152	7.666∼16.224	<0.001
Pulmonary function index							
**FVC (%)**							
<80	88 (9.8)		1.000			1.000	
≥80	811 (90.2)	0.618	0.361∼1.058	0.079	0.529	0.344∼0.814	0.004
FEV_1_ (%)							
<80	166 (18.5)		1.000			1.000	
≥80	733 (81.5)	0.660	0.421∼1.036	0.071	0.635	0.441∼0.916	0.015
FEV_1/_FVC (%)							
<70	155 (17.2)		1.000			1.000	
≥70	744 (82.8)	0.790	0.478∼1.307	0.359	0.785	0.526∼1.173	0.237

BMI, Body Mass Index; ECOG PS, Eastern Cooperative Oncology Group Performance Status; FVC, Forced vital capacity; FEV_1_, Forced expiratory volume in the first second.

#### Correlation analysis based on histological features

A total of 597 patients (66.4%) underwent lobectomy as the primary surgical protocol, 184 patients (20.5%) underwent segmentectomy, and 112 patients (12.5%) underwent cuneiform resection. As of the 8th edition of the international TNM staging system for lung cancer, 78.6% of patients were in stage I, and most of the histological types showed adenocarcinoma without lymph node metastasis.

Univariate analysis based on tumor histological features showed a better prognosis after segmentectomy and wedge-shaped resection. Patients with lobectomy, tumor diameter ≥2.25, and non-adenocarcinoma had a worse prognosis. Compared with NSCLC patients with a Number of lymph nodes removed surgically (LNs) <10, patients with 10–19 LNs had a higher postoperative survival rate. In addition, pathological stage, T stage, and N stage are important factors affecting the prognosis of NSCLC patients. See **Table 2**. In the PFS correlation analysis, segmental resection and wedge resection were the improvement factors for PFS, while lobectomy and total pulmonary resection were the risk factors for disease progression. Tumor location in the middle lobe of the right lung, tumor diameter, LNs, histological type, and TNM stage all affect patient prognosis (**Table 2**).

**Table 2:** Univariate Cox regression analysis based on histological features.

Category	n (%)	Overall Survival			Progression Free Survival		
		*HR*	*95% CI*	*P - value*	*HR*	*95% CI*	*P - value*
Tumor location							
Superior lobe of left lung	222 (24.7)		1.000			1.000	
Inferior lobe of left lung	148 (16.5)	0.694	0.365∼1.325	0.269	0.796	0.487∼1.301	0.363
Superior lobe of right lung	293 (32.6)	1.413	0.774∼2.580	0.261	1.054	0.632∼1.757	0.840
Middle lobe of right lung	79 (8.8)	0.643	0.353∼1.170	0.148	0.613	0.379∼0.994	0.047
Inferior lobe of right lung	157 (17.5)	1.274	0.606∼2.680	0.522	1.341	0.754∼2.383	0.318
Pulmonary lobectomy							
No	302 (33.6)		1.000			1.000	
Yes	597 (66.4)	4.461	2.155∼9.236	<0.001	5.555	3.075∼10.037	<0.001
Segmentectomy							
No	715 (79.5)		1.000			1.000	
Yes	184 (20.5)	0.167	0.053∼0.530	0.002	0.086	0.027∼0.269	<0.001
Wedge resection							
No	787 (87.5)		1.000			1.000	
Yes	112 (12.5)	0.244	0.077∼0.771	0.016	0.239	0.098∼0.582	0.002
Total pneumonectomy							
No	893 (99.3)		1.000			1.000	
Yes	6 (0.7)	2.469	0.607∼10.037	0.207	4.555	1.685∼12.314	0.003
Tumor diameter (cm)							
<2.25	645 (71.7)		1.000			1.000	
≥2.25	254 (28.3)	4.524	2.989∼6.849	<0.001	5.546	3.957∼7.773	<0.001
Number of lymph node dissection							
<10	433 (48.2)		1.000			1.000	
10∼19	380 (42.3)	3.220	1.847∼5.611	<0.001	3.237	2.022∼5.181	<0.001
≥20	86 (9.6)	1.421	0.897∼2.249	0.134	1.782	1.234∼2.575	0.002
Histological type							
Adenocarcinoma	793 (88.2)		1.000			1.000	
Non-adenocarcinoma	106 (11.8)	2.362	1.478∼3.773	<0.001	2.499	1.710∼3.651	<0.001
Differentiated degree							
Poorly differentiated	101 (11.2)		1.000			1.000	
Moderately differentiated	676 (75.2)	0.242	0.109∼0.536	<0.001	0.141	0.066∼0.303	<0.001
High differentiation	122 (13.6)	0.401	0.252∼0.637	<0.001	0.370	0.255∼0.537	<0.001
Pathological stage							
Stage I	707 (78.6)		1.000			1.000	
Stage II	87 (9.7)	106.771	46.118∼247.190	<0.001	104.645	59.948∼182.667	<0.001
Stage III	105 (11.7)	31.486	13.033∼76.066	<0.001	26.923	14.999∼48.326	<0.001
T-staging							
T_1_	544 (60.5)		1.000			1.000	
T_2_	314 (34.9)	19.450	8.270∼45.745	<0.001	41.306	20.460∼83.388	<0.001
T_3_	29 (3.2)	8.058	3.808∼17.052	<0.001	11.142	6.006∼20.670	<0.001
T_4_	12 (1.3)	3.693	2.250∼6.063	<0.001	5.155	3.426∼7.757	<0.001
N-staging							
N_0_	743 (82.6)		1.000			1.000	
N_1_	59 (6.6)	75.332	25.123∼225.879	<0.001	92.986	35.517∼243.449	<0.001
N_2_	92 (10.2)	40.410	23.350∼69.936	<0.001	60.493	38.577∼94.859	<0.001
N_3_	5 (0.6)	13.078	6.907∼24.762	<0.001	20.647	12.599∼33.834	<0.001

#### Correlation analysis based on pathological features and immunohistochemistry

Among the patients with genetic mutations, 561 (62.4%) were EGFR gene mutation positive; the P53 gene mutation was positive in 682 cases (75.9%). In the expression of Ki-67, 76.9% of patients showed low expression. Vascular invasion, intraductal tumor thrombus, lymphatic vessel invasion, and nerve invasion were observed in 62.8%, 11.0%, 61.4%, and 2.7%, respectively.

Univariate Cox regression analysis based on pathological and immunohistochemical characteristics showed that, compared with patients with negative P53 expression, wild-type patients had a worse prognosis. Patients with ≥25% Ki-67 expression were risk factors for OS compared with patients with <25% Ki-67 expression. In addition, it was observed that patients with intravascular embolic tumors and nerve invasions had shorter postoperative survival. See **Table 3**. In the PFS correlation analysis, patients with wild-type P53 gene mutation, Ki-67 expression ≥25%, vascular invasion, intraductal tumor thrombin, lymphatic vessel invasion, and nerve invasion were more likely to develop disease progression (**Table 3**).

**Table 3:** Univariate Cox regression analysis based on pathological features and immunohistochemistry.

Category	n (%)	Overall Survival			Progression Free Survival		
		*HR*	*95% CI*	*P - value*	*HR*	*95% CI*	*P - value*
P53							
Negative	217 (24.1)		1.000			1.000	
Mutant type	236 (26.3)	1.141	0.664∼1.958	0.633	1.303	0.828∼2.051	0.252
Wild type	446 (49.6)	1.905	1.089∼3.332	0.024	2.238	1.405∼3.565	0.001
EGER							
Negative	338 (37.6)		1.000			1.000	
Positive	561 (62.4)	1.121	0.722∼1.742	0.610	1.308	0.918∼1.864	0.138
Ki-67 (%)							
<25	691 (76.9)		1.000			1.000	
25∼49	87 (9.7)	3.140	1.932∼5.103	<0.001	2.422	1.606∼3.654	<0.001
≥50	121 (13.5)	3.065	1.853∼5.068	<0.001	3.259	2.168∼4.899	<0.001
Vascular invasion							
No	334 (37.2)		1.000			1.000	
Yes	565 (62.8)	1.293	0.849∼1.970	0.231	1.536	1.080∼2.185	0.017
Endovascular thrombus							
No	800 (89.0)		1.000			1.000	
Yes	99 (11.0)	3.691	2.389∼5.702	<0.001	5.197	3.696∼7.308	<0.001
lymphatic vessel invasion							
No	347 (38.6)		1.000			1.000	
Yes	552 (61.4)	1.283	0.848∼1.940	0.239	1.589	1.121∼2.251	0.009
Perineural invasion							
No	875 (97.3)		1.000			1.000	
Yes	24 (2.7)	3.559	1.725∼7.342	0.001	3.262	1.716∼6.202	<0.001

#### Correlation analysis based on complex indexes of inflammation and nutrition

The fibrinogen/albumin ratio (FAR), systemic immune-inflammatory index (SII), fibrinogen to prealbumin ratio (FPR), neutrophil/lymphocyte ratio (NLR), alkaline phosphatase to prealbumin ratio (APR), pan-immune inflammatory value (PIV), C-reactive protein/albumin ratio (CAR), platelet/lymphocyte ratio (PLR), Red blood cell distribution width/albumin ratio (RAR), lymphocyte/monocyte ratio (LMR), Prognostic nutritional index (PNI), serum free fatty acid/albumin ratio (FFA/Alb), serum albumin globulin ratio (AGR), advanced lung cancer inflammation index (ALI) and albumin to alkaline phosphatase ratio (AAPR) were calculated by the formula. and the best cutoff values of the complex indexes of inflammation and nutrition were obtained by area under the curve (AUC) and converted into binary variables (**Table 4**). Cox univariate analysis showed that inflammation and nutrition complex indexes were prognostic factors for OS and PFS. As shown in **Table 5**.

**Table 4:** Calculation methods and cut-off values of inflammation and nutrition complex indexes.

Variate	Computational Methods	Overall Survival				Progression Free Survival			
		cutoff value	AUC	*95% CI*	*P value*	cutoff value	AUC	*95% CI*	*P value*
**FAR**	FIB/Alb	0.079	0.655	0.598∼0.712	<0.001	0.085	0.652	0.603∼0.700	<0.001
**SII**	Plt × ANC/Lym	487.097	0.629	0.574∼0.685	<0.001	464.723	0.602	0.554∼0.651	<0.001
**FPR**	FIB/PA	0.013	0.624	0.564∼0.684	<0.001	0.013	0.631	0.582∼0.681	<0.001
**NLR**	ANC/Lym	1.642	0.622	0.566∼0.678	<0.001	1.451	0.602	0.554∼0.651	<0.001
**APR**	ALP/PA	0.335	0.612	0.552∼0.673	<0.001	0.340	0.602	0.549∼0.655	<0.001
**PIV**	ANC × Plt × Mono/Lym	156.175	0.607	0.551∼0.663	0.001	111.537	0.596	0.548∼0.644	<0.001
**CAR**	CRP/Alb	0.017	0.599	0.539∼0.660	0.001	0.017	0.594	0.542∼0.645	<0.001
**PLR**	Plt/Lym	143.733	0.571	0.509∼0.634	0.021	145.371	0.558	0.506∼0.611	0.025
**RAR**	RDW/Alb	1.131	0.564	0.506∼0.622	0.039	1.131	0.566	0.514∼0.618	0.011
**LMR**	Lym/Mono	4.169	0.447	0.385∼0.509	0.087	4.843	0.429	0.379∼0.479	0.006
**PNI**	Alb+5 × Lym	45.510	0.437	0.374∼0.500	0.043	45.535	0.466	0.413∼0.518	0.184
**FFA/Alb**	FFA/Alb	9.812	0.427	0.362∼0.492	0.018	8.003	0.463	0.408∼0.518	0.151
**AGR**	Alb/Glb	1.485	0.409	0.348∼0.470	0.003	1.151	0.426	0.374∼0.478	0.004
**ALI**	BMI×Alb/NLR	70.061	0.374	0.320∼0.428	<0.001	70.516	0.398	0.349∼0.446	<0.001
**AAPR**	Alb/ALP	0.045	0.359	0.298∼0.421	<0.001	0.044	0.394	0.340∼0.447	<0.001

FAR, The fibrinogen/albumin ratio; SII, Systemic immune-inflammatory index; FPR, fibrinogen to prealbumin ratio; NLR, Neutrophil/lymphocyte ratio; APR, Alkaline phosphatase to prealbumin ratio; PIV, Pan-immune inflammatory value; CAR, C-reactive protein/albumin ratio; PLR, platelet/lymphocyte ratio; RAR, Red blood cell distribution width/albumin ratio; LMR, lymphocyte/monocyte ratio; PNI, Prognostic nutritional index; FFA/Alb, serum free fatty acid/albumin ratio; AGR, serum albumin globulin ratio; ALI, advanced lung cancer inflammation index; AAPR, albumin to alkaline phosphatase ratio.

**Table 5:** Univariate Cox regression analysis based on complex indexes of inflammation and nutrition.

Category	Overall Survival				Progression Free Survival			
	n (%)	*HR*	*95% CI*	*P - value*	n (%)	*HR*	*95% CI*	*P - value*
Inflammatory complex index								
NLR								
Below	424 (47.2)		1.000		327 (36.4)		1.000	
Above	475 (52.8)	2.529	1.604∼3.987	<0.001	572 (63.6)	2.176	1.471∼3.218	<0.001
PLR								
Below	641 (71.3)		1.000		649 (72.2)		1.000	
Above	258 (28.7)	2.062	1.378∼3.084	<0.001	250 (27.8)	1.675	1.202∼2.334	0.002
SII								
Below	611 (68.0)		1.000		592 (65.9)		1.000	
Above	288 (32.0)	2.511	1.685∼3.742	<0.001	307 (34.1)	1.949	1.413∼2.688	<0.001
LMR								
Below	408 (45.4)		1.000		547 (60.8)		1.000	
Above	491 (54.6)	0.572	0.383∼0.856	0.007	352 (39.2)	0.562	0.392∼0.804	0.002
PIV								
Below	426 (47.4)		1.000		240 (26.7)		1.000	
Above	473 (52.6)	2.151	1.398∼3.311	<0.001	659 (73.3)	2.372	1.495∼3.764	<0.001
Nutrient complex index								
FAR								
Below	527 (58.6)		1.000		614 (68.3)		1.000	
Above	372 (41.4)	2.545	1.683∼3.848	<0.001	285 (31.7)	2.538	1.839∼3.504	<0.001
FPR								
Below	627 (69.7)		1.000		594 (66.1)		1.000	
Above	272 (30.3)	2.073	1.391∼3.090	<0.001	305 (33.9)	2.133	1.546∼2.943	<0.001
RAR								
Below	606 (67.4)		1.000		606 (67.4)		1.000	
Above	293 (32.6)	1.713	1.147∼2.559	<0.001	293 (32.6)	1.699	1.229∼2.350	0.001
AGR								
Below	528 (58.7)		1.000		100 (11.1)		1.000	
Above	371 (41.3)	0.527	0.337∼0.825	0.005	799 (88.9)	0.379	0.258∼0.556	<0.001
ALI								
Below	592 (65.9)		1.000		596 (66.3)		1.000	
Above	307 (34.1)	0.271	0.148∼0.496	<0.001	303 (33.7)	0.393	0.257∼0.600	<0.001
PNI								
Below	198 (22.0)		1.000		199 (22.1)		1.000	
Above	701 (78.0)	0.460	0.302∼0.700	<0.001	700 (77.9)	0.541	0.384∼0.764	<0.001
AAPR								
Below	368 (40.9)		1.000		341 (37.9)		1.000	
Above	531 (59.1)	0.425	0.280∼0.644	<0.001	558 (62.1)	0.505	0.366∼0.698	<0.001
CAR								
Below	386 (42.9)		1.000		385 (42.8)		1.000	
Above	513 (57.1)	2.287	1.432∼3.652	0.001	514 (57.2)	2.112	1.465∼3.044	<0.001
APR								
Below	547 (60.8)		1.000		555 (61.7)		1.000	
Above	352 (39.2)	1.878	1.256∼2.809	0.002	344 (38.3)	1.947	1.410∼2.688	<0.001
FFA/Alb								
Below	431 (47.9)		1.000		300 (33.4)		1.000	
Above	468 (52.1)	0.541	0.351∼0.833	0.005	599 (66.6)	0.676	0.488∼0.936	0.018

FAR, The fibrinogen/albumin ratio; SII, Systemic immune-inflammatory index; FPR, fibrinogen to prealbumin ratio; NLR, Neutrophil/lymphocyte ratio; APR, Alkaline phosphatase to prealbumin ratio; PIV, Pan-immune inflammatory value; CAR, C-reactive protein/albumin ratio; PLR, platelet/lymphocyte ratio; RAR, Red blood cell distribution width/albumin ratio; LMR, lymphocyte/monocyte ratio; PNI, Prognostic nutritional index; FFA/Alb, serum free fatty acid/albumin ratio; AGR, serum albumin globulin ratio; ALI, advanced lung cancer inflammation index; AAPR, albumin to alkaline phosphatase ratio.

### Multivariate Cox analysis

The multivariable Cox regression model of OS included factors that were statistically different from each other on their own. The results showed that pathological stage and neuroinvasion were significant factors for poor survival (p<0.05). High serum free fatty acid/serum albumin ratio (HR: 0.576, 95% CI: 0.344–0.962, p = 0.035) was an independent prognostic factor for OS improvement. As shown in **Table 6**. The Kaplan-Meier survival curve constructed based on the above factors is shown in **Supplementary Figure S1**.

**Table 6:** Multivariate Cox analysis of overall survival and progression-free survival.

Factor	Overall Survival			Progression Free Survival		
	*HR*	*95%CI*	*P - value*	*HR*	*95%CI*	*P - value*
Perineural invasion						
Without vs. With	2.568	1.013∼6.512	0.047			
Pathological stage						
Stage I		1.000			1.000	
Stage II	46.731	6.832∼319.651	<0.001	29.655	6.914∼127.191	<0.001
Stage III	16.964	4.276∼67.295	<0.001	7.971	2.913∼21.813	<0.001
FFA/Alb						
<9.812 vs. ≥9.812	0.576	0.344∼0.962	0.035	0.655	0.430∼0.998	0.049
Smoking status						
Never vs. Current/past				2.052	1.121∼3.754	0.020
Adjuvant radiation therapy						
No vs. Yes				3.501	2.136∼5.739	<0.001
LNs						
<10					1.000	
10∼19				0.481	0.255∼0.909	0.024
≥20				0.646	0.402∼1.037	0.070
T stage						
T_1_					1.000	
T_2_				2.551	0.968∼6.719	0.058
T_3_				2.508	1.093∼5.752	0.030
T_4_				1.939	1.139∼3.303	0.015
N stage						
N_0_					1.000	
N_1_				3.760	0.656∼21.561	0.137
N_2_				3.013	0.879∼10.332	0.079
N_3_				2.558	1.128∼5.803	0.025
Tumor location						
Superior lobe of left lung					1.000	
Inferior lobe of left lung				0.530	0.288∼0.975	0.041
Superior lobe of right lung				0.847	0.450∼1.596	0.607
Middle lobe of right lung				0.613	0.335∼1.120	0.112
Inferior lobe of right lung				0.757	0.375∼1.527	0.437

FFA/Alb, serum free fatty acid/albumin ratio; LNs, Number of lymph nodes removed surgically.

Multivariate Cox analysis of PFS showed that smoking history, adjuvant radiotherapy, pathological stage, T stage, and N stage were significant factors for disease progression (p<0.05). Left inferior lobe, LNs 10-19, and a high serum free fatty acid/serum albumin ratio were prognostic factors for improvement of PFS (**Table 6**). The Kaplan-Meier survival curve constructed based on the above factors is shown in **Supplementary Figure S2**.

### Establishment and verification of nomogram

#### Visualization of a nomogram of OS

**Figure 1:**
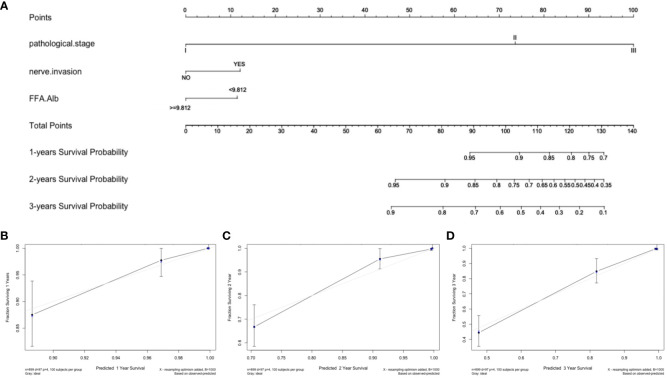
Nomogram and calibration curve for predicting overall survival of NSCLC patients. **(A)** Nomogram model; Calibration curves for 1-year **(B)**, 2-year **(C)**, and 3-year **(D)** Overall Survival.

**Figure 2:**
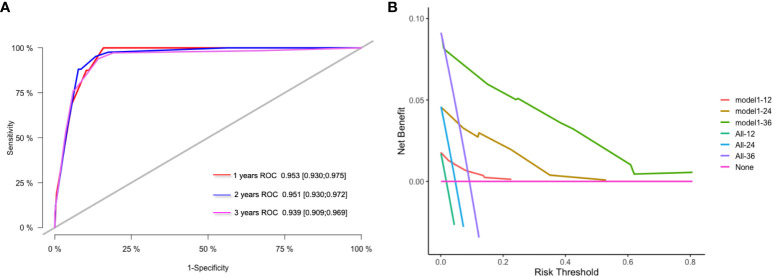
ROC curve and DCA curve of prediction model. **(A)** ROC curves for 1 -, 2 - and 3-year survival predictions; **(B)** 1-year, 2-year and 3-year clinical value DCA curves.

The model’s predictions were shown visually using R software to look at individualized prognostic predictions for patients with surgically treated NSCLC using variables that were statistically significant in Cox regression analysis. These variables included pathological stage, neuroinvasion, and the FFA/Alb ratio. See **Figure 1A**. The score of the first row corresponding to the vertical of each index is added to obtain the total score, which can intuitively determine the estimated survival probability of patients in 1 year, 2 years, and 3 years. The higher the score, the worse the predicted prognosis. The prediction performance of the nomogram model was evaluated by C-index and calibration curve, and the results showed that the model predicted OS with a C-index of 0.927 (95%CI: 0.907-0.947). The Bootstrap self-sampling method (B = 1000) was used to internally verify the prediction model. The predicted survival rate was taken as the horizontal coordinate and the actual survival rate as the vertical coordinate. The calibration curve showed that there was good consistency between the predicted survival rate and the actual observation probability of NSCLC patients after 1, 2, and 3 years. It shows that the model fits well. See **Figures 1B-D**. ROC curves for 1, 2, and 3-year survival rates were plotted according to independent factors to evaluate the accuracy of the model. The results showed that the area under curve (AUC) of the model was 0.953 (95% CI: 0.930–0.975) and 0.951 (95% CI: 0.930), respectively. 0.930-0.9972), 0.939 (95% CI: 0.909-0.969), and the model showed good differentiation (**Figure 2A**). Decision curve analysis (DCA) was used to evaluate the application value of the model, and the results showed that when the threshold probability was greater than 0.05, the threshold probability was positively correlated with the net benefit level of the model, as shown in **Figure 2B**. Dynamic nomogram: https://qq1586541381.shinyapps.io/DynNomapp/.

#### Visualization of a nomogram of PFS

The nomogram of PFS showed the location of the tumor, its pathological stage (T stage, N stage), the patient’s smoking history, the number of lymph nodes that were surgically removed, and the FFA/Alb ratio (**Supplementary Figure S3A**). This information was used to figure out the patient’s individual risk of the disease getting worse. The nomogram model predicts that the C index of PFS is 0.944 (95% CI: 0.922–0.966). Calibration curve analysis results show that the model fits well (**Supplementary Figure S3B-3D**). ROC curves of disease progression at 1, 2, and 3 years were plotted according to independent factors to evaluate the accuracy of the model (**Supplementary Figure S4A**). The results showed that the AUC of the model was 0.9530.952 (95% CI: 0.925–0.979) and 0.951 (95% CI: 0.925), respectively (0.916–0.985) and 0.939 (95% CI: 0.913–0.965). DCA results show that the model has high clinical application value(**Supplementary Figure S4B**). Dynamic nomogram: https://sxs1097213689.shinyapps.io/DynNomapp/.

## Discussion

By integrating multiple risk factors to predict survival prognosis and assessing the cumulative impact of all prognostic factors, the nomograph can effectively estimate the probability of survival at a specific point in time, and the practicality and convenience of this method in the field of lung cancer research have been widely recognized. Cai et al. (16)developed a nomogram model to predict cancer-specific survival in patients diagnosed with stage IA non-small cell lung cancer. Zuo et al. (17)developed a prediction model for stage IB NSCLC patients, while Jia et al. (18)constructed a nomogram model to predict mortality in NSCLC surgery patients, utilizing histological and pathological features as the primary variables. The resulting C-index for lung cancer-related death was 0.73 (95%CI, 0.72-0.74). Zhang et al. (19)developed a nomogram model to depict the correlation between tumor size and survival. Sun et al. (20)constructed a survival prediction model specifically for elderly patients with early NSCLC after undergoing surgery. Additionally, Xie et al. (21)established a model to forecast the postoperative survival duration of patients based on preoperative peripheral blood indicators. Nevertheless, the predictive models utilized in the past were constrained by the restrictions inherent in the SEER database as well as the patient baseline characteristics, which restricted the inclusion of all pertinent prognostic markers. Furthermore, the researchers have observed that the C-index values of the nomogram models they developed are all below 0.8. This suggests that the prediction performance of these models is not considerably superior to that of the TNM evaluation method (22). The objective of our study is to expand the scope of parameters considered, encompassing patient baseline characteristics, histological features, pathological and immunohistochemical findings, and inflammatory and nutritional markers. This comprehensive analysis aims to identify factors that influence postoperative survival and disease progression in patients with operable NSCLC. Additionally, we seek to develop a more dependable nomogram model for predicting postoperative survival in NSCLC patients.

The multi-parameter nomographic model shows that the pathological stage is the best predictor of OS and PFS. Many studies support the idea that people with stage I lung cancer can have a 3-year survival rate of more than 90% and people with stage II lung cancer can have a 3-year survival rate of about 70%. The 3-year survival rate for stage III lung cancer patients is usually less than 50%. Compared with patients diagnosed with stage I lung cancer, stage II and III patients have a larger tumor diameter and local lymph node metastasis, which leads to an increased likelihood of disease progression and thus affects survival prognosis (4, 5). Second, as our column chart shows, the FFA/Alb ratio is another common factor affecting both OS and PFS. Fatty acids, usually in free form or as components of triglycerides, phospholipids, and cholesterol, play important roles in energy storage, signal transduction, and gene transcription regulation (23). In normal tissues, *de novo* synthesis of fatty acids is limited to fat and liver cells. However, in order to meet their own high metabolic requirements and adapt to the reduction of sero-derived fatty acids in the tumor microenvironment, tumor cells will increase the expression of fatty acid synthesis-related enzymes to enhance fatty acid synthesis. Therefore, the enhancement of fatty acid anabolism is considered to be one of the important markers of tumors (24). In addition, due to the characteristics of rapid metabolism and proliferation of tumor cells, nutritional status indicators such as albumin are also important clinical prognostic parameters for evaluating lung cancer treatment. Malnutrition has been observed in previous studies to be associated with poorer overall survival and time to tumor progression in lung cancer patients (25, 26). It is important to note that anti-cancer treatments, including surgery, may exacerbate the severity of malnutrition. Second, malnutrition was associated with an increased susceptibility to perioperative death. Therefore, it is essential to incorporate nutritional assessment into the pre-treatment regimen for cancer patients. Studies have shown that providing nutritional support can effectively reduce the adverse effects of malnutrition on perioperative prognosis (27, 28).

Perineural invasion (PNI) is an important factor affecting the postoperative survival of patients with NSCLC. A study by Demir et al. (29)found that the presence of PNI had a significant negative impact on 3-year and 5-year survival (3-year survival decreased from 54% to 32% and 5-year survival decreased from 15% to 0%). Kilicgun et al. (30)found that patients diagnosed with stage IA lung cancer accompanied by nerve invasion had a lower survival rate than those diagnosed with stage IIIA without nerve invasion. In addition, another retrospective study also confirmed this finding, and the 3-year survival rate of stage I NSCLC patients with and without PNI was 23.3% and 63.2%, respectively (31). Our results showed that the presence of PNI was associated with a 2.24-fold increase in the risk of death, and PNI still had good predictive value after excluding confounding factors through multivariate analysis.

FFA/Alb ratio. The results of this study confirmed that different primary sites of tumors had certain effects on the disease progression of patients, and the risk levels were as follows: middle lobe of right lung > upper lobe of left lung > lower lobe of left lung > lower lobe of right lung > upper lobe of right lung. Right middle lobe tumors have long been considered to have a worse prognosis than tumors at other sites. The study by Vincent et al. (32)found that 19 patients who underwent tumor removal had a median survival time of only 9.6 months, compared to 14.6 to 25.6 months for those whose tumors were located elsewhere. The study by Freise et al. (33)found that the 5-year survival rate for lung cancer patients who had the right middle lobe surgically removed was 18%. In contrast, the 5-year survival rate for patients whose tumors were located in other lung lobes was 26.4% to 34.3%. This phenomenon may be related to the presence of a large number of lymphatic drainage points in the right middle lobe extending into the upper and lower mediastinal regions. Our study did not find a statistical difference between tumor location and OS, but there is a potential association with PFS, which may be related to the distribution of lymph node metastasis. The study by Yang et al. (34)analyzed the association between mediastinal lymph node metastasis distribution and survival in patients with operable NSCLC (≤3 cm). The results showed that the upper right lobe had 4 stations (17.7%); the right middle lobe had 7 stations (14.9%); the lower right lobe had 7 stations (19.8%); the left upper lobe had 7 stations (16.6%); and the left upper lobe had 5 stations (18.2%). Guo et al. (35)studied the association between the main site and mediastinal lymph node location in patients with radical resection of N2 lymph node metastasis, and the results showed that the highest rate (100%) was at 2/4 stations, which occurred in the right upper lobe. The proportion of 7 stations in the right middle lobe/lower lobe was relatively high, accounting for 80% and 88.9%, respectively. In addition, lymph node removal is an important part of surgery. In this study, LNs<10 is a risk factor for prognosis, which is consistent with the existing literature (36, 37). This correlation may be attributed to the fact that the number of lymph nodes cleared during surgery is correlated with the disease stage to a certain extent. On the other hand, patients with fewer intraoperative lymph node dissections may have undetected lymph node metastases, leading to inadequate treatment (38, 39). Smoking status is also considered a potential risk factor for disease progression. Cheng et al. (40)found that smoking has the potential to accelerate tumor progression, a phenomenon attributed to the smoking-induced accumulation of M2-tumor-associated macrophages (M2-TAMs) around non-small cell lung cancer tissue. In addition, radiation therapy is considered to be one of the most effective methods of tumor control. In a recent study on the correlation between off-target radiotherapy and tumor metastasis, it was found that the occurrence of tissue damage was associated with an increased susceptibility to metastasis risk, and the reason was closely related to neutrophils. During tissue repair, local neutrophils are activated, creating a better environment for metastatic cancer cells and inadvertently promoting tumor metastasis, which has been verified in mouse models (41).

Based on each risk factor, the postoperative survival and disease progression of patients with operable NSCLC were constructed, and the C-index was 0.927 (907–0.947) and 0.944 (0.922–0.966), respectively, indicating high predictive performance. In order to minimize overfitting, the Bootstrap method (repeated sampling 1 000 times) was adopted for internal verification, and the model was evaluated by a calibration curve. The model showed a good degree of differentiation and calibration, indicating a high degree of consistency between the predicted survival probability and the actual observed probability. The results of the DCA curve show that the model has good clinical effectiveness. However, there are still some limitations to this study. First, this was a single-center retrospective and non-randomized study. Second, this study lacks external validation to evaluate the model, so more multi-center studies are needed to improve the model in the future.

## Conclusion

This study conducted a multi-factor assessment of patients based on their baseline characteristics, histological features, pathological and immunohistochemical results, inflammation, and nutritional indicators and established a nomogram model that can be used as a practical and reliable tool to predict postoperative survival and disease progression in patients with NSCLC.

## Data availability statement

The raw data supporting the conclusions of this article will be made available by the authors, without undue reservation.

## Ethics statement

The studies involving humans were approved by Ethics Committee of Qingdao Municipal Hospital. The studies were conducted in accordance with the local legislation and institutional requirements. Written informed consent for participation was not required from the participants or the participants’ legal guardians/next of kin in accordance with the national legislation and institutional requirements.

## Author contributions

SM: Formal analysis, Investigation, Methodology, Software, Validation, Visualization, Writing – original draft, Writing – review & editing. LW: Conceptualization, Methodology, Project administration, Resources, Supervision, Validation, Visualization, Writing – original draft, Writing – review & editing.
