# Supplementary material for: Prognostic factors and predictive model construction in patients with non-small cell lung cancer: a retrospective study
Source: Front Oncol. 2024 May 24;14:1378135. doi: 10.3389/fonc.2024.1378135 (PMC11157049; doi:10.3389/fonc.2024.1378135)
Supplement: Supplementary file 1 [file DataSheet_1.docx]

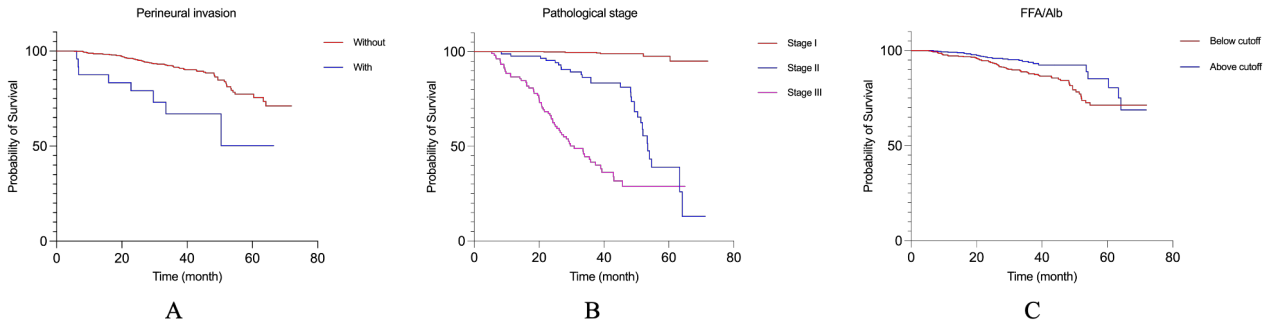


**Figure S1. The Kaplan-Meier survival curve based on OS multifactor regression**

1. **Perineural invasion; (B) Pathological stage; (C) FFA/Alb**


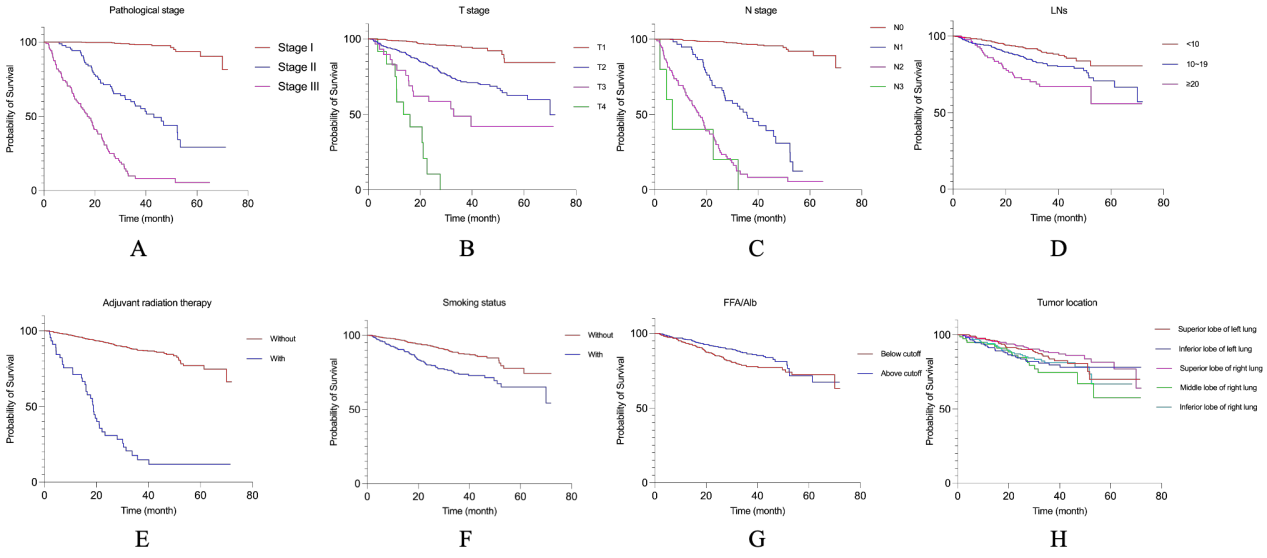


**Figure S2. The Kaplan-Meier survival curve based on PFS multifactor regression**

1. **Pathological stage; (B) T stage; (C) N stage; (D) LNs; (E) Adjuvant radiation therapy; (F) Smoking status; (G) FFA/Alb; (H) Tumor location**


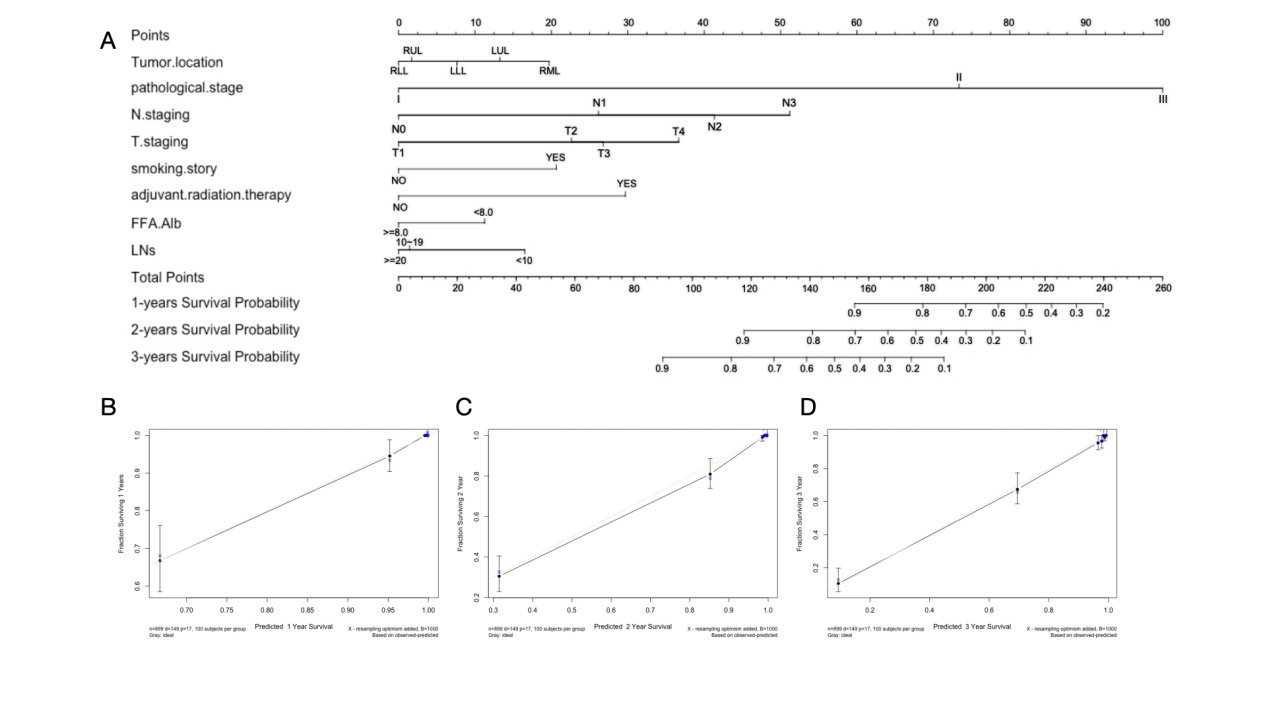


**Figure S3. Nomogram and calibration curve for predicting progression-free survival of NSCLC patients**

1. **Nomogram model; Calibration curves for 1-year (B), 2-year (C), and 3-year (D) progression-free survival**

**
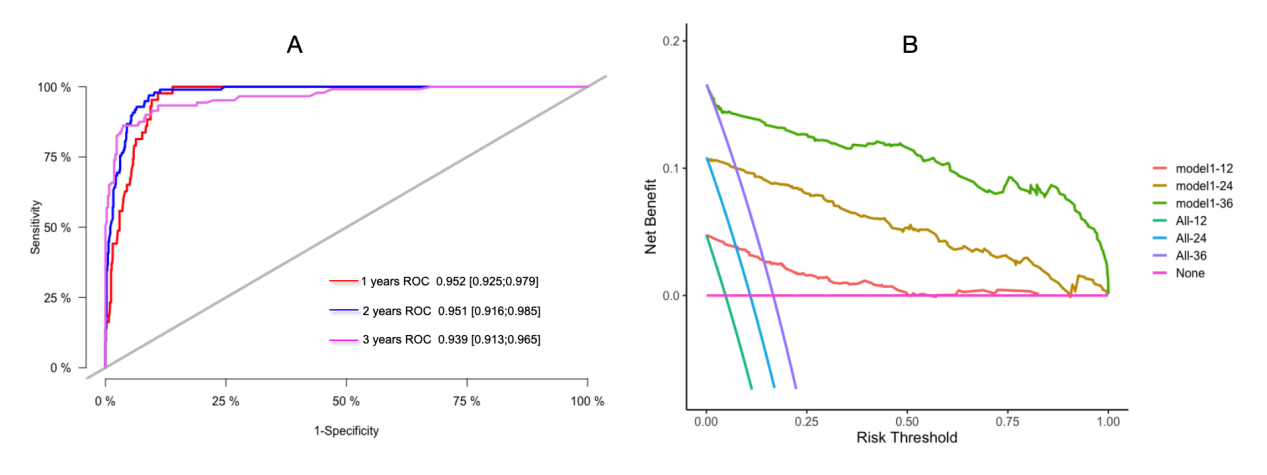
**

**Figure S4. ROC curve and DCA curve of prediction model**

**(A) ROC curves for 1 -, 2 - and 3-year Progression Free Survival predictions; (B) 1-year, 2-year and 3-year clinical value DCA curves**
